# Mitral Valve Infective Endocarditis Associated With Prednisolone-Induced Immunosuppression: A Case Report

**DOI:** 10.7759/cureus.48474

**Published:** 2023-11-07

**Authors:** Tomohiro Nakajima, Yutaka Iba, Tsuyoshi Shibata, Akihito Ohkawa, Nobuyoshi Kawaharada

**Affiliations:** 1 Cardiovascular Surgery, Sapporo Medical University, Sapporo, JPN

**Keywords:** mitral valve regurtitation, mitral valve, immunosupressions, predonisolon, infective endocarditis

## Abstract

A 74-year-old man with pemphigoid, for which he was on a daily regimen of 14 mg of prednisolone and immunosuppressive drugs, was admitted to the orthopedic surgery department with a fever of 38 °C. An MRI scan of his head revealed multiple bilateral cerebral infarcts, and echocardiography showed a 30-mm structure attached to the anterior apex of the mitral valve. The patient was diagnosed with infective endocarditis and administered antibiotic therapy. Five days after the diagnosis, the patient underwent mitral valve surgery, during which the mitral valve was observed to be severely deteriorated and hence replaced with a bioprosthetic valve. Blood flow disturbance was observed in the right lower extremity, and a thrombectomy was performed. A dispersed vegetation around the heart was observed and removed. After the surgery, the patient progressed without mediastinitis and had a good postoperative course. He was discharged from the hospital on the 56th postoperative day after continued antibiotic therapy.

## Introduction

Patients on steroids or immunosuppressive drugs are sometimes treated with open heart surgery. The risk of postoperative infection is high in these patients, and hence it is advisable to lower the dosage of the drugs as much as possible before performing the operation. However, it is often difficult to make such a decision because a sudden reduction in steroid dosage may cause a flare-up of the underlying disease. We report a case of a patient who was taking steroids and immunosuppressive drugs for pemphigoid and developed infective endocarditis of the mitral valve and required early surgery due to embolic symptoms. The steroid dose was reduced as quickly as possible, and surgery was subsequently performed.

## Case presentation

**Figure 1:**
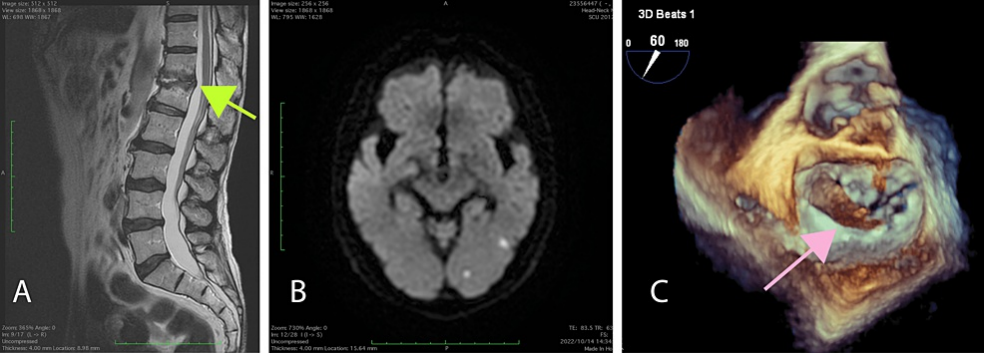
Preoperative findings (A) Preoperative lumbar MRI. Destruction of the first lumbar vertebrae was observed, and pyogenic spondylitis was suspected. The green arrow shows the fractured site. (B) Brain MRI. Diffusion MRI showed multiple infarction sites. (C) Transesophageal echocardiogram. 30-mm-sized vegetation was detected at the mitral valve anterior commissure. The pink arrow shows the vegetation MRI: magnetic resonance imaging

A 74-year-old male with a history of treatment-resistant pemphigus presented to our hospital with complaints of fever (38 °C) for two days and back pain. He was on 14 mg of steroids and immunosuppressive drugs daily for pemphigoid. Laboratory results indicated a C-reactive protein level of 20 mg/dL. A spine MRI showed pyogenic spondylitis, and the results of a spinal tap confirmed methicillin-susceptible Staphylococcus aureus infection (Figure 1A). Meanwhile, he was started on intravenous penicillin G (12,000 units/day). A brain MRI revealed multiple infarcts in the bilateral cerebral cortex, which were thought to be dispersed vegetation (Figure 1B), though the patient had no neurological symptoms before surgery. After a thorough systemic examination, echocardiography revealed severe mitral regurgitation and a 30-mm-sized vegetation adhering to the anterior leaflet, leading to a diagnosis of infective endocarditis (Figure 1C). His dosage of penicillin G was increased to 24,000 units/day.

**Figure 2:**
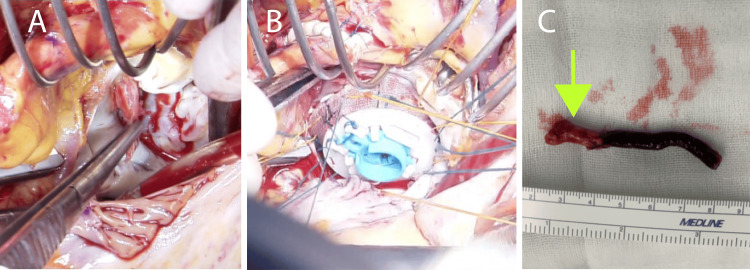
Operative findings (A) Vegetation was attached to the mitral valve anterior commissure. (B) Mitral valve replacement with MITRIS RESSILA 29 mm. (C) Vegetation and thrombosis. MSSA was cultured at the vegetation (green arrow) MSSA: methicillin-susceptible Staphylococcus aureus

Even though the patient had multiple embolic symptoms, surgery was deemed high-risk on account of his 14-mg prednisolone regimen. After conferring with dermatology and cardiology, we reduced the prednisolone dosage to 5 mg daily to lower the risk of mediastinitis. A full-body contrast-enhanced CT was performed preoperatively, which showed no evidence of embolization in the spleen or kidneys. Surgery was performed under cardiac arrest on his 10th day in the hospital. During surgery, we observed vegetation attached to the anterior commissure to the anterior apex of the mitral valve (Figure 2A). After excising it, an edge-to-edge repair was attempted, but the valve became detached due to infection. Mitral valve replacement (MITRIS RESSILA 29 mm, Edwards Lifesciences, Irvine, CA) was then performed (Figure 2B). We also detected a right lower-limb ischemia, and hence a thrombectomy was conducted. Embolic decompensation was assumed to be scattered vegetation (Figure 2C). The operation time was 430 minutes, the aortic clamp time was 221 minutes, and the extracorporeal circulation time was 270 minutes. Antegrade myocardial protection was performed, and thereafter, cardiac arrest was maintained with retrograde coronary perfusion once every 25 minutes.

**Figure 3:**
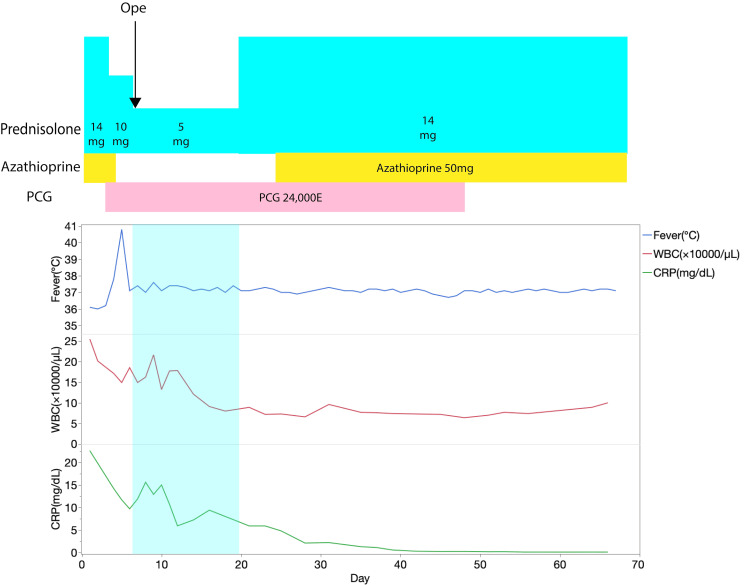
Perioperative chart After the patient was diagnosed with infective endocarditis, we reduced the dose of prednisolone gradually, stopped azathioprine, and started intravenous penicillin G (PCG) infusion. We conducted mitral valve replacement with the patient on 5-mg prednisolone. The patient was maintained on 5 mg of prednisolone for two weeks postoperatively, and azathioprine was resumed in the third postoperative week. PCG was administered for six weeks according to the guidelines

Figure 3 shows the perioperative course. Prednisolone was maintained at 5 mg for two weeks, including the day of surgery. Penicillin G was administered for six weeks postoperatively according to guidelines [1]. There was no fever, and both his white blood cell and C-reactive protein levels decreased. The patient progressed without mediastinitis and had a good postoperative course. He was discharged home on postoperative day 56. The patient followed up at the outpatient clinic every three months after the surgery. At the nine-month follow-up, no recurrence of infection or pemphigoid was detected.

## Discussion

Infective endocarditis is a rare, systemic, septicemic disease that can invade the valve leaflets, endocardium, and large-vessel endomembrane, resulting in bacteremia, vascular embolization, cardiac injury, and even death [2,3]. Long-term steroid use and immunosuppressive therapy may increase the risk of postoperative infectious complications [4]. However, it is unclear whether steroid reduction can control infection, and there is a risk that the underlying primary disease will flare up after tapering [5]. Nevertheless, to reduce the risk of postoperative infection, the dose of steroids and immunosuppressive therapies should be reduced before surgery.

In this case, infective endocarditis was diagnosed, and intravenous penicillin G was started immediately. When mitral valve replacement surgery was deemed necessary, the patient’s steroid dose was tapered to 5 mg. Azathioprine was discontinued via dose reductions every three days. During surgery, dispersed vegetation was found in the right lower extremity and removed. The patient recovered uneventfully and there were no signs of spreading infection, which is a risk associated with the steroid reduction [6]. In the absence of mediastinitis, the steroid dose was increased again to 14 mg. One week later, azathioprine was restarted. The patient did not develop any postoperative infection.

## Conclusions

We reported a case of infective endocarditis in a patient on a long-term regimen of 14 mg of daily prednisolone. Postoperatively, the patient developed an embolism in the lower extremity, probably caused by vegetative growth, but no other major complications or infections occurred. In our patient on oral immunosuppressive drugs, a serious complication was avoided by reducing the dosage of such drugs.

## References

[1] Nakatani S, Ohara T, Ashihara K (2019). JCS 2017 Guideline on prevention and treatment of infective endocarditis. Circ J.

[2] Rajani R, Klein JL (2020). Infective endocarditis: a contemporary update. Clin Med (Lond).

[3] Li J, Ruegamer T, Brochhausen C (2022). Infective endocarditis: predictive factors for diagnosis and mortality in surgically treated patients. J Cardiovasc Dev Dis.

[4] Pai KR, Ramnarine IR, Grayson AD, Mediratta NK (2005). The effect of chronic steroid therapy on outcomes following cardiac surgery: a propensity-matched analysis. Eur J Cardiothorac Surg.

[5] Dvirnik N, Belley-Cote EP, Hanif H (2018). Steroids in cardiac surgery: a systematic review and meta-analysis. Br J Anaesth.

[6] Grapsa J, Blauth C, Chandrashekhar YS, Prendergast B, Erb B Jr, Mack M, Fuster V (2022). Staphylococcus aureus infective endocarditis: JACC patient pathways. JACC Case Rep.

